# The epidemiology and surveillance response to pandemic influenza A (H1N1) among local health departments in the San Francisco Bay Area

**DOI:** 10.1186/1471-2458-13-276

**Published:** 2013-03-27

**Authors:** Wayne TA Enanoria, Adam W Crawley, Winston Tseng, Jasmine Furnish, Jeannie Balido, Tomás J Aragón

**Affiliations:** 1 Division of Epidemiology, University of California at Berkeley, Berkeley, California, USA; 2 Center for Infectious Diseases and Emergency Readiness, University of California at Berkeley, Berkeley, California, USA; 3 Health Research for Action, University of California at Berkeley, Berkeley, California, USA; 4, San Francisco Department of Public Health, San Francisco, California, USA

**Keywords:** Influenza A (H1N1), Epidemiology, Surveillance, Public health preparedness, Public health emergency response

## Abstract

**Background:**

Public health surveillance and epidemiologic investigations are critical public health functions for identifying threats to the health of a community. Very little is known about how these functions are conducted at the local level. The purpose of the Epidemiology Networks in Action (EpiNet) Study was to describe the epidemiology and surveillance response to the 2009 pandemic influenza A (H1N1) by city and county health departments in the San Francisco Bay Area in California. The study also documented lessons learned from the response in order to strengthen future public health preparedness and response planning efforts in the region.

**Methods:**

In order to characterize the epidemiology and surveillance response, we conducted key informant interviews with public health professionals from twelve local health departments in the San Francisco Bay Area. In order to contextualize aspects of organizational response and performance, we recruited two types of key informants: public health professionals who were involved with the epidemiology and surveillance response for each jurisdiction, as well as the health officer or his/her designee responsible for H1N1 response activities. Information about the organization, data sources for situation awareness, decision-making, and issues related to surge capacity, continuity of operations, and sustainability were collected during the key informant interviews. Content and interpretive analyses were conducted using ATLAS.ti software.

**Results:**

The study found that disease investigations were important in the first months of the pandemic, often requiring additional staff support and sometimes forcing other public health activities to be put on hold. We also found that while the Incident Command System (ICS) was used by all participating agencies to manage the response, the manner in which it was implemented and utilized varied. Each local health department (LHD) in the study collected epidemiologic data from a variety of sources, but only case reports (including hospitalized and fatal cases) and laboratory testing data were used by all organizations. While almost every LHD attempted to collect school absenteeism data, many respondents reported problems in collecting and analyzing these data. Laboratory capacity to test influenza specimens often aided an LHD’s ability to conduct disease investigations and implement control measures, but the ability to test specimens varied across the region and even well-equipped laboratories exceeded their capacity. As a whole, the health jurisdictions in the region communicated regularly about key decision-making (continued on next page) (continued from previous page) related to the response, and prior regional collaboration on pandemic influenza planning helped to prepare the region for the novel H1N1 influenza pandemic. The study did find, however, that many respondents (including the majority of epidemiologists interviewed) desired an increase in regional communication about epidemiology and surveillance issues.

**Conclusion:**

The study collected information about the epidemiology and surveillance response among LHDs in the San Francisco Bay Area that has implications for public health preparedness and emergency response training, public health best practices, regional public health collaboration, and a perceived need for information sharing.

## Background

Surveillance and epidemiologic investigations are critical public health functions for identifying threats to the health of a community. In an emergency, public health surveillance is “the ongoing systematic collection, analysis, interpretation, and management of public health-related data to verify a threat or incident of public health concern, and to characterize and manage it effectively through all phases of the incident” [1].

Once an event has been detected, public health departments conduct epidemiologic investigations to confirm that there is, indeed, a health threat in the community, identify the source of the disease, injury, or exposure, and describe the characteristics of its occurrence with respect to time, place, and persons affected. Health departments collect this information to determine which control measures to implement to most effectively prevent further illnesses, injuries, or exposures. Thus, timely public health surveillance and epidemiologic investigations are crucial activities for an effective public health response because they allow public health officials to gather health information, prioritize response activities, and make appropriate public health decisions.

Although public health surveillance and epidemiologic investigations are important, very little is known about how these functions are conducted at the local level. Public health professionals who conduct epidemiologic functions at the local level may belong to diverse professional disciplines and have varying levels of formal training in epidemiology [2]. Epidemiologic investigations during a public health emergency are conducted through networks of public health professionals and other partners [3], but how these networks work during an emergency is not well understood.

Previous research studies have demonstrated a recent decline in the epidemiology capacity of local and state health departments across the nation. According to national surveys conducted by the Council of State and Territorial Epidemiologists (CSTE), the epidemiology capacity among state health departments declined by 12% from 2004 to 2009, especially in program areas such as bioterrorism and emergency response [4]. State health departments in the United States needed an additional 1,490 epidemiologists in 2009. Other research studies have found that, from 2001 to 2007, state health departments had increasing levels of responsibility, such as providing additional preventative services and oversight of hospitals and other institutions. These studies also found that all emerging practice areas, such as bioterrorism preparedness, injury control and prevention, tobacco control and prevention, and environmental epidemiology had expanded [5]. Despite the decline in epidemiology capacity, public health surveillance and epidemiologic investigations continue to be important and expanding functions for responding to public health threats in our communities [6], and they constitute one of the fifteen public health preparedness capabilities for state and local public health preparedness activities [1].

### The emergence of novel influenza A (H1N1) in 2009

On April 17, 2009, the Centers for Disease Control and Prevention (CDC) determined that infection with a novel influenza A (H1N1) virus caused two cases of febrile respiratory illness occurring in children in southern California [7]. Subsequently, cases of human infection with the same strain of novel influenza A (H1N1) were reported in Mexico, Canada, and other countries [8]. On April 20, 2009, the 61 local health departments (LHDs) in California along with the California Department of Public Health (CDPH) initiated enhanced surveillance for hospitalized and fatal cases of pandemic influenza A (H1N1) because there was a lack of information at that time about the severity of illness, clinical features of infection, and the populations at risk for complications from pandemic influenza A (H1N1) infection [9]. Because this new strain of swine influenza A (H1N1) had not circulated in humans previously, there was concern that a large proportion of the population would be susceptible to infection and that seasonal influenza vaccine would not provide protection.

The purpose of the Epidemiology Networks in Action (EpiNet) Study was to describe the epidemiology and surveillance response to the 2009 pandemic influenza A (H1N1) by city and county health departments in the San Francisco Bay Area in California, as well as document lessons learned from the response, in order to strengthen future public health preparedness and response planning efforts in the region.

## Conceptual framework

### The epidemiology and surveillance response to pandemic influenza A (H1N1)

The ability to diagnose and investigate health problems in the community is one of the ten essential public health services [10]. Epidemiology skills and capacity play an important role in describing the impact of a health threat, measuring its magnitude, and developing interventions and testing their effectiveness. Thus, local and state public health agencies collect public health surveillance data, draw epidemiologic inferences from the data, and make decisions for public health action based on these inferences (so called “data for action”[6]).

**Figure 1:**
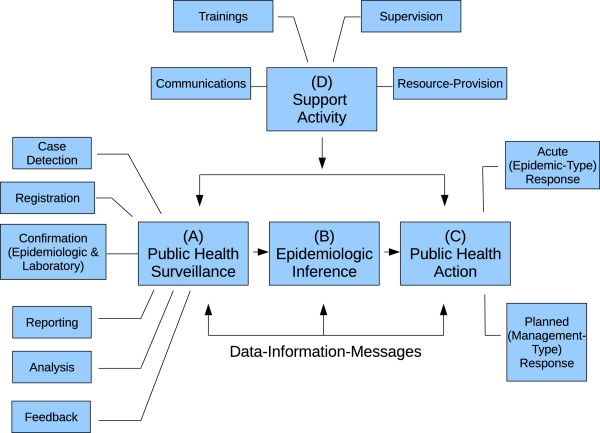
**Conceptual framework of public health surveillance and public health action.** The figure was adapted from McNabb et al. [11] and modified to emphasize the importance of epidemiologic inferences.

An effective public health surveillance and epidemiology response requires the cohesive interplay between many public health activities. Using a conceptual framework published by McNabb et al. [11], which describes the interdependent processes of public health surveillance and public health action in the context of health sector reform, we modified the framework to emphasize the role and importance of epidemiologic inference in taking public health action (Figure 1). The conceptual framework describes six public health surveillance activities (Figure 1, (A) Public Health Surveillance): (1) case detection; (2) the collection of specific descriptive characteristics of each case, which are entered or registered into a public health record (registration); (3) confirmation of the cases through the evaluation of epidemiologic criteria and/or laboratory results; (4) reporting of the cases from lower levels of the public health system to higher ones; (5) analyses of the case information; and (6) flow of information from the higher levels of the public health system back down to the lower ones (feedback). After the data are interpreted (Figure 1, (B) Epidemiologic Inference), public health actions (Figure 1, (C) Public Health Action) can take two forms: (1) acute or epidemic-type responses, which include immediate public health actions such as epidemiologic investigations, contact investigations, and targeted interventions, or (2) planned or management-type responses, such as community public health education, purchasing of immunization supplies, and re-allocating public health personnel and resources in response to changing disease trends. In this conceptual framework, four support activities (Figure 1, (D) Support Activity) are designed to improve the core functions: communications, training, supervision, and resource-provision. Thus, public health surveillance and public health actions can be viewed as interdependent processes that relate to one another through inflow and outflow of data, information, and messages.

Local health departments are decision-making entities that take actions based on information in their environments [12]. Like other organizations, they consist of a network of components (i.e., people, resources, knowledge, and tasks) and the relations among these components [13]. An organization adapts to situations by altering the set of people, resources, knowledge, tasks, or the relations among these components to achieve its objectives. Like other emergency response organizations, an LHD responds and coordinates through social networks, which has implications for its ability to coordinate during a response [3]. Like other emergency response personnel, public health professionals act within the limits of available information [14] – information that must be communicated in a timely manner by external agents – to inform how they will respond in a dynamic, changing environment [15]. Public health personnel collect information within their health jurisdiction in order to determine what is happening in any situation, but may also use external sources beyond their jurisdiction in order to maintain broader situation awareness. As a result, it is crucial to understand with whom public health personnel communicated, both within and outside of their organizations, in order to maintain situation awareness during the pandemic.

This study focused on the four components of the public health system (i.e., the mission, structural capacity, processes, and outcomes [16]) that were involved in these activities, rather than the entire public health system. During any infectious disease emergency, local and state health departments have many key objectives, which include identifying any threats to the health of communities and preventing future morbidity and mortality from occurring (see section List of Public Health objectives in an emergency).

### Public health objectives in an emergency

Objectives

1. Detect the incident. 

• Advance warning enables the system to respond sooner.

2. Define the incident. 

• Describe the situation in terms of person, place, and time.

3. Define the at-risk population. 

• Target limited resources.

4. Measure the magnitude of the incident. 

• Obtain valid data on needs and health status of the affected community.

• Guide response efforts in terms of providing needed services.

5. Forecast the incident and predict future impact. 

• Anticipate future morbidity and mortality.

• Anticipate the at-risk populations.

• Anticipate future needs for resources and health care.

6. Develop interventions and test their effectiveness. 

• Alleviate the burden of impact of the incident on the health of individuals and their communities.

7. Monitor the ongoing situation. 

• Guide response efforts if the situation changes.

## Methods

### Study population

**Figure 2:**
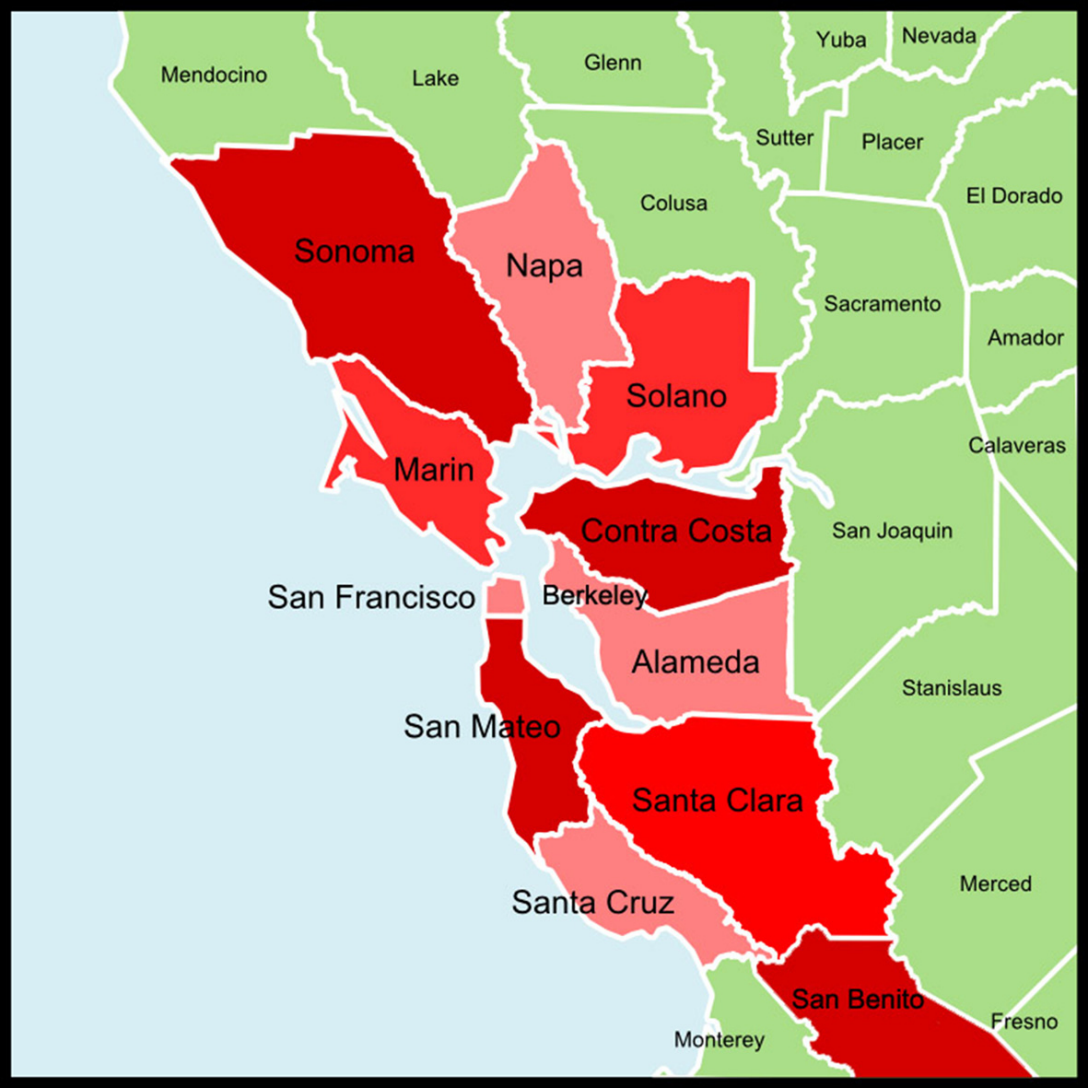
The Association of Bay Area Health Officials.

The Association of Bay Area Health Officials (ABAHO) is a group of health officers and health directors from thirteen city and county health departments in the San Francisco Bay Area that meets on a regular basis to discuss important public health issues [17]. In November 2006, ABAHO convened a Pandemic Influenza Planning Work Group to increase collaboration for regional pandemic influenza planning. This group consisted of representatives from twelve cities and counties in the San Francisco Bay Area: Alameda, City of Berkeley, Contra Costa, Marin, Napa, San Benito, San Francisco, San Mateo, Santa Clara, Santa Cruz, Solano, and Sonoma [18]. In order to understand the strengths and weaknesses of this regional network of LHDs, the EpiNet study focused its investigation on the twelve cities and counties that participated in the ABAHO Pandemic Influenza Planning Work Group since 2006 (now called the ABAHO Public Health Preparedness Subcommittee). The twelve LHDs are shown in Figure 2 and the populations they serve are summarized in Table 1.

**Table 1:** ABAHO cities and counties

**Local health department**	**2009 Population estimate**^ † ^
Alameda county	1,491,482
City of Berkeley^*†*^	101,555
Contra Costa County	1,041,274
Napa County	134,650
Marin County	250,750
San Benito County	55,058
City & County of San Francisco	815,358
San Mateo County	718,989
Santa Clara County	1,784,642
Santa Cruz County	256,218
Solano County	407,234
Sonoma County	472,102

^*‡*^The 2009 population estimate is according to the U.S. Census Bureau.

^*†*^The City of Berkeley is within the jurisdictional boundaries of Alameda County.

The study was approved by the Committee for the Protection of Human Subjects at the University of California at Berkeley.

### Recruitment of key informants

In order to recruit individuals into the study, one of the investigators (TJA) sent an email to health officers of the local health jurisdictions informing them of the study’s goals and objectives. The health officers identified the appropriate persons within their organizations to be interviewed. The research staff scheduled interviews with individuals who responded and consented to participate. In addition, a presentation was given at an ABAHO Public Health Preparedness Subcommittee meeting in order to inform additional public health professionals in the region about the study. Members of the Public Health Preparedness Subcommittee identified additional key informants for the study.

### Data collection

In order to understand how each local health jurisdiction conducted its epidemiology and surveillance activities, research staff (WE, AC, and JB) conducted key informant interviews with public health professionals at two different levels within each organization: (1) epidemiologists and/or public health professionals responsible for the epidemiology and surveillance activities in response to novel influenza A (H1N1), and (2) health officers or their designees who were responsible for overseeing the response to H1N1 (e.g., deputy health officers). Semi-structured key informant interviews were conducted from September 2010 to March 2011, administered by an interviewer to direct the discussions. The number of respondents interviewed per organization ranged from one to three, with an average of two respondents per organization. Most interviews lasted between 60 and 90 minutes. Written informed consent was obtained from each participant at the start of the interview.

During the interviews, key informants were asked about their organization’s structure, case and contact investigations, data sources for situation awareness, laboratory testing, other public health emergencies or outbreaks that may have occurred during the H1N1 response, and networks of public health professionals utilized during the response. Through interviews with representatives from the local health jurisdictions, information about day-to-day operations as well as decision-making considerations involved in the public health response was collected. The key informant interviews were recorded and the audio files were transcribed. The study staff conducted a total of 23 interviews with key informants among eleven ABAHO cities and counties. Each key informant was offered a ¡DOLLAR/¿50 VISA gift card for participating in the study. The twelfth local health jurisdiction declined to participate in interviews but instead completed a written questionnaire adapted from the original key informant interview guide. For the purpose of reporting results, this organization’s responses have been aggregated along with the other organizations’ responses where appropriate.

Additional documents pertaining to the organizations’ epidemiology and surveillance response activities (e.g., After Action Reports (AARs), surveillance reports, data analysis summaries, etc.) were collected and reviewed as needed. The review of additional documents served to corroborate statements made during the interviews as well as to obtain a more in-depth understanding of each organization’s actions during the response.

### Data analysis

Content and interpretive analyses were performed on all interview transcripts to identify key themes and concepts as described by the key informants. A preliminary codebook was developed from the questions included in the key informant interview guides and the descriptive coding of the first four transcripts reviewed (two epidemiologist and two health officer transcripts). Descriptive coding for the development of the preliminary codebook was conducted by two research team members (AC, JF). The coding between the two research team members was compared for consistency followed by group discussions with the full research team (AC, JF, WT, and WTAE). Coding of additional transcripts was conducted by one individual (AC), and new codes were developed throughout the coding process, as they emerged from the data. After all of the transcripts were reviewed, a complete codebook of descriptive codes and family codes was completed and finalized. Codes were grouped independently into families and sub-families. Independent groups were compared and all families and sub-families were created through discussion. Codes were created and analyzed using ATLAS.ti software.

## Results

The information obtained in the key informant interviews is summarized in this section by the main topics: disease investigation and surge capacity, use of the Incident Command System (ICS) to manage the public health response, epidemiologic data sources for situation awareness, laboratory capacity for novel influenza A (H1N1) testing, and regional communication. Table 2 provides a summary of the 23 respondents’ titles, average years served in their organization, and the average number of personnel directly supervised by the respondents.

**Table 2:** Respondent roles in 2009

**Respondent title**	**Number of**	**Average years in**	**Average number of**
	**respondents**	**organization**	**personnel supervised**
Supervising epidemiologist	3	6	3
Epidemiologist	7	6	0
Emergency services specialist	1	18	0
Health officer	5	16	4
Health officer/director	3	11	6
Deputy health officer	4	8	6

### Disease investigations and surge capacity

The EpiNet study focused heavily on the initial response to the emergence of the H1N1 pandemic, including the high level of disease investigation activities that occurred in the spring and summer of 2009. During the initial phase of the pandemic all cases of laboratory-confirmed novel influenza A (H1N1) were reported to CDPH, including all influenza-related hospitalizations and deaths. Beginning July 15, 2009, LHDs were asked to report only hospitalizations, fatalities and outbreaks of novel H1N1 influenza to CDPH; reporting of individual outpatient cases was no longer required [19]. On August 12, 2009, LHDs were asked to report hospitalized cases of novel H1N1 influenza as weekly aggregate numbers. Intensive care unit cases and fatal cases continued to be reported using individual case report forms [20]. These reporting guidelines provided by CDPH were adhered to by each LHD in the EpiNet study, although many participants noted that the rapidly-changing guidelines resulted in “information overload”. 

“There was a flood of information sent out, more than we could keep up with initially. Just keeping track of daily reports and changing advice...felt like a full time job all by itself.”

In order to comply with these reporting guidelines, case investigations were conducted during the initial wave of the H1N1 pandemic by all 12 LHDs in the EpiNet study. Investigations were conducted to identify the source of the H1N1 influenza virus, facilitate timely diagnoses, implement control measures, and aid in the characterization of the virus. The type of staff members involved in case investigations varied by LHD and included public health nurses, disease investigators, epidemiologists, and health officers. In many cases, the number of staff normally assigned to disease investigation tasks was not sufficient to handle the volume of case and contact investigations at the outset of the pandemic. In these instances, additional staff were brought in either to assist directly with disease investigations or to backfill positions being vacated by those involved in the investigations. These additional staff, referred to as “surge capacity staff”, included nurses from sections other than communicable disease control, staff from some agencies’ tuberculosis or sexually-transmitted disease units, and/or recently retired public health employees. An LHD’s ability to recruit additional staff from within its agency depended largely on the support of the agency’s leadership, pre-existing plans and procedures, and resources available to the particular LHD. In addition to obtaining surge staff from within their LHD, some agencies reported receiving assistance from external sources. One LHD received assistance from a CDC Epidemic Intelligence Service Officer, another reported obtaining support through its jurisdiction’s Medical Reserve Corps (MRC), and yet another described receiving assistance from other public agencies in its jurisdiction.

While some surge staff were familiar with the activities involved in their new assignments, many were unfamiliar with communicable disease control and epidemiology functions and required Just-in-Time Training (JITT) in conducting case and contact investigations, as well as in using personal protective equipment, such as N95 respirators. Ten of the twelve LHDs reported providing some level of training for surge staff. 

“In an urgent or emergency situation or a situation like this in which the DOC is expanded, then we have a need to bring more people in, so we brought in other public health nurses who may or may not have received specific training in this area, but certainly got JITT training and were given their...job descriptions and their objectives and the tools that they needed to assist with the work. And so that was fairly successful, but at the same time it was also challenging because it required pulling people from their normal day-to-day work...which you know, brings up the importance of continuity of operations planning for the entire department.”

Further emphasizing the need for surge capacity staff was the fact that many respondents reported other infectious disease outbreaks during the H1N1 pandemic, including tuberculosis, pertussis, and measles, as well as more “routine” outbreaks, such as norovirus. While the “routine” events were not reported to have had an impact on continuity of operations for most departments, cases of measles and tuberculosis required a more significant response. 

“Previously when we had a measles case, we had done an activation for those and done isolation and quarantine and it was extensive. But we couldn’t do all of those activities. We couldn’t activate for that when we’re activated for flu....Part of that is trying to think carefully about how you’re going to use public health resources. Do we need this level of follow-up based on ’XYZ’ situation? We definitely had less of a response and part of that was because of resources.”

Given limited resources, organizations had to prioritize public health activities and, in some cases, delayed day-to-day tasks in order to handle the H1N1 response. Twelve of the 23 respondents specifically indicated that, to varying degrees, other tasks were put aside. 

“I think that the continuity was really on a very limited basis. Of course we... took all our reports and followed through, but it was really on a very limited basis... throughout the entire agency we were focused on H1N1. We have a continuity of operations plan on paper but it wasn’t, it really wasn’t implemented.”

### Use of the incident command system to manage the public health response

While disease investigations were an important component of LHDs’ early response to the H1N1 pandemic, many other staff, including emergency preparedness managers and laboratorians also played important roles. In order to effectively manage key staff from a variety of sections, including the surge capacity staff providing additional support for key functions, all 12 LHDs in the EpiNet study implemented ICS. Yet, while the implementation of this system was universal, the manner in which ICS was implemented and utilized varied among the LHDs. For instance, while most health departments indicated that ICS was employed continuously throughout the response period, two health departments reported activating ICS in two separate phases. The initial phase focused on situation awareness and characterization of the epidemiology of novel influenza A (H1N1) infection, while the second phase focused on vaccine distribution after the vaccine became available.

Only one agency reported strict adherence to the ICS structure and reporting guidelines. It was far more common for LHDs to use the ICS structure and reporting process intermittently, often reverting to their day-to-day structure and reporting for managing the response. Some agencies employed a modified version of ICS to suit their organizational needs (such as combining the Logistics and Finance sections). For some agencies, this was a matter of convenience, while others admitted that knowledge of ICS among staff members was inadequate, stating that “our own use of ICS was clumsy because we don’t have a broadly held understanding of ICS, including at the highest levels [of the organization]”.

The study found that agencies that used ICS to manage previous events, such as seasonal influenza or tuberculosis investigations, were able to utilize staff with ICS experience, while other LHDs with less experience in ICS needed “Just-in-Time Training” during the pandemic. The LHDs that utilized ICS for responding to other events prior to novel influenza A (H1N1) were able to make use of lessons learned from previous ICS activations to adjust their ICS response to the pandemic, resulting in smoother implementation during the response. 

“One of the things that we’ve changed is that...we’re taking every opportunity to use real events to exercise the use of ICS and the DOC [Departmental Operations Center] and the emergency response structure....I think it’s much more engaging for people and helps us learn a lot better if we treat actual occurrences that might not really necessitate the use of ICS or the DOC, but treat them as exercises to rev up all of that, to reinforce our use of [ICS] and to practice using it so that we are more adept at it if we really need to do it.”

Respondents provided specific insights into some of the obstacles to implementing ICS, including: (1) staff were assigned to ICS roles that did not align with their skill sets or job functions (for example, an epidemiologist being assigned the lead for the Logistics section); (2) staff within the ICS structure often reported to staff other than their day-to-day supervisors, complicating communication efforts; (3) there were barriers to obtaining the necessary permissions for staff to access data as part of their ICS duties; (4) staff responsible for critical response functions were burdened by additional ICS leadership responsibilities; and (5) LHDs struggled with where to place epidemiology and surveillance staff and functions within the ICS organizational chart. Two LHDs placed epidemiologists within the Planning section of their ICS structure, while the other ten LHDs placed epidemiologists within the Operations section. One respondent (an epidemiologist) from an LHD that placed epidemiologists within the Operations section stated that, in the future, the organization would prefer to place epidemiologists in the Planning section in order to help directly inform the development of the Incident Action Plan, a critical function of the Planning section. 

“You know, we tried to pass information [to help the development of the Incident Action Plan] along to the Planning section but I think there’s been a lot of confusion where to place the epidemiologists within the [ICS organizational] chart. I keep going back and forth and as a result of this response, I think they’re going to place me back in with Plans.”

The above quote highlights the importance of epidemiology functions within the ICS structure. Public health staff carrying out epidemiology and surveillance functions were tasked with managing a number of data sources to inform situation awareness and often aided key-decision makers in translating the data into actionable recommendations.

### Epidemiologic data sources for situation awareness

The key informants in the study identified numerous data sources used for situation awareness during the pandemic (Table 3). The data collection efforts were highly dependent upon the external relationships that had been developed prior to or during the pandemic by each LHD. Coordination and communication between LHDs and CDPH, area hospitals, clinics, long-term care facilities, schools, and other organizations were important for disease surveillance, particularly in the early stages of the pandemic. Local health departments leveraged partnerships with community organizations and businesses in their jurisdictions to collect information and communicate effectively about the H1N1 pandemic.

**Table 3:** Epidemiologic data sources for situation awareness

**Type of data**	**Number of local health departments**
Case reports	12
Hospitalized/Fatal cases	12
Laboratory data	12
School absenteeism	11
Emergency room census	9
ILI sentinel surveillance	5
Vital statistics data	5
Hospital bed count	4
Workplace absenteeism	2
911 Calls/Logs	2
Other syndromic surveillance	2

Abbreviations: ILI, influenza-like illness.

All health jurisdictions utilized case reports, reporting of hospitalized and fatal cases, and laboratory test results to inform their understanding of the H1N1 pandemic and to guide their response. Hospital data (including information on co-morbidities), demographic data, and laboratory data were all reported as being particularly useful for characterizing the pandemic because they were reliable data sources. One respondent explained that medical data, as opposed to other syndromic data sources, were the most useful and consistent. 

“There are a lot of ways you can try to do flu surveillance and epi but the only one that really works is through clinic care, through the medical community. Because no one else anywhere has an obligation to report anything or an incentive to report anything or a system designed to make it possible to report anything.”

Nine LHDs captured emergency room or outpatient data that were usually characterized by chief complaint or presenting symptoms. LHDs also made use of influenza-like illness “sentinels” that reported outpatient data to CDPH; vital statistics, such as the number of deaths from pneumonia and influenza; and hospital bed counts. A minority of the LHDs in the study also utilized workplace absenteeism rates, 911 calls, medical dispensing data, poison control center calls, and airport surveillance.

Eleven LHDs utilized school absenteeism data, with varying degrees of success. Nine respondents discussed specific challenges related to school absenteeism data which, for most LHDs, were a newly acquired data source. Respondents explained that absenteeism data rarely included information about the specific illness causing the reported absence and that data collection systems among school districts were not standardized and difficult to aggregate; in many cases absenteeism data systems were not automated and required manual collection, entry, and transfer of data in order to obtain. 

“School reporting is really tricky. We have eleven school districts in the county and I wouldn’t say that each one has its own system, but there are many different systems used by the different districts for tracking attendance and some of them can give reasonably frequent data on student absences and student enrollment from which you can calculate percent absent. Some of them, but not very many, get information about whether absence is due to illness or not. Most of them don’t.”

While LHDs obtained data from a number of sources in order to monitor the H1N1 pandemic, there were instances in many organizations when not all of the desired data were obtainable. One respondent wanted additional outpatient data from private health care providers because the health department did not have influenza sentinel surveillance data for its jurisdiction. Other data that were sought included more complete school absenteeism data, length of stay data for hospitalized patients, workplace absenteeism data, and the number of negative influenza test results from the public health laboratory in order to provide a denominator to accompany the number of positive tests reported for novel H1N1.

### Laboratory capacity for novel influenza A (H1N1) testing

As noted earlier, the study found that laboratory data were particularly valuable sources of information for LHDs during the pandemic response. The lack of capacity to test influenza specimens at the local level using reverse-transcriptase polymerase chain reaction (RT-PCR) negatively affected case and contact investigations and slowed LHDs’ ability to determine the status of suspected influenza cases, which in turn slowed the implementation of mitigation efforts.

The CDPH Viral and Rickettsial Disease Laboratory (VRDL) obtained the necessary reagents for subtyping the 2009 influenza A (H1N1) virus early in the pandemic, but these reagents were only available to some LHDs later. Of the twelve health jurisdictions interviewed, eight LHDs reported having the capability to test influenza specimens for novel influenza A (H1N1) to the level of “Influenza A (unsubtypable)” during the pandemic (it should also be noted that two of these eight LHDs shared a single, jointly-operated public health laboratory). Among these eight LHDs, three reported obtaining the necessary reagents during the course of the pandemic to subtype influenza specimens. The four LHDs without influenza testing capabilities either sent their specimens to the state laboratory for testing (two LHDs) or utilized a neighboring jurisdiction’s laboratory (two LHDs). All jurisdictions reported submitting specimens to the VRDL at CDPH for additional testing and virus characterization, as required by evolving guidance issued by CDC during the pandemic.

At least one health department reported that, despite being equipped with a laboratory for influenza testing, the capacity of the laboratory was exceeded during the initial phase of the pandemic, when testing was being performed on a high volume of specimens. 

“We had so many [specimens], the lab, it was like trying to...get a fire hydrant’s worth of water through a pipette...I mean it was just ridiculous.”

Another challenge reported by respondents included laboratories lacking appropriate systems to prioritize the testing of certain specimens. It was noted by some jurisdictions that health care providers were either not testing patients for influenza often enough or were relying too heavily on rapid diagnostic influenza tests, which have been reported to have lower sensitivity to detect 2009 H1N1 virus in upper respiratory specimens when compared to real-time RT-PCR [21]. 

“I really think that until the tests improve that we’re going to be limited in our surveillance. As I mentioned, we had a really hard time convincing providers not to do the rapid test, which is a terrible test for H1N1. [There was a] real resistance to using nasopharyngeal swabs [among providers].”

Despite the challenges faced by many public health laboratories during the pandemic, regional support, in the form of shared public health laboratories, testing provided by neighboring health jurisdictions, and support from CDPH’s VRDL helped to alleviate some of the pressure faced by LHDs in the Bay Area.

### Regional communication among the Association of Bay Area Health Officials

Laboratory testing was not the only area in which LHDs collaborated on a regional level during the pandemic. The members of ABAHO convened regular conference calls to discuss each LHD’s efforts to control novel H1N1. Topics of discussion included school closures, the use of antivirals and personal protective equipment, vaccine priority groups, and vaccine distribution. Interviews with health officers, deputy health officers, and health directors confirmed the value that the majority of participants found in these regional discussions. As one health officer explained, 

“I wouldn’t miss an ABAHO call if I could help it...[T]alking to people who are making decisions that are going to happen in the county next to you is, without exception, an incredibly important thing to do.”

Another health officer noted the value of understanding one’s peers’ perspectives, even if they differed from one’s own. 

“...I think just talking to people who know the field, are smart – that’s just really helpful. It’s different perspectives and it’s okay they had different perspectives because I don’t think any of us know the right answers, but it was just helpful to kind of hear other people’s perspectives and either cement your opinion or have your opinion changed.”

The ABAHO members discussed a number of key decisions related to influenza mitigation. During the H1N1 pandemic, each of the LHDs established new (or made use of existing) relationships with schools in its jurisdiction. With rapidly-changing guidance on school closures from CDC [22] and little scientific evidence of effectiveness to rely on, many health officers were tasked with deciding whether or not to close schools in an effort to slow the spread of H1N1 in their communities. While CDC initially recommended closing schools for up to 14 days when a laboratory-confirmed case of influenza was detected, they soon changed the policy to focus on keeping sick individuals out of schools and childcare facilities for the duration of their illness[23]. One health officer described the situation and their communication with other health officers in the ABAHO network, 

“...ABAHO did also work with the state and maybe also with CDC about change in the school closure guidance early on... There was a lot of really extreme and early recommendations out there that some people felt very compelled to follow. And then CDC was reviewing it and deciding to change it, and CDPH was deciding to change it. And we’re like, ’guys, we’re out here, we’ve got schools’... So we were kind of in this awkward place where the formal recommendation says you have to do this but everybody thinks you actually don’t.”

Ultimately, five of the twelve LHDs made the decision to close schools in an attempt to slow the spread of novel influenza A (H1N1) in their jurisdictions before the CDC changed their recommendation regarding school closure.

Many respondents valued knowing what approaches neighboring counties were taking regarding issues such as control measures, vaccine distribution, and even public messaging, even if this knowledge did not change their own decisions regarding these issues for their jurisdictions. In particular, three health officers noted the importance of the ABAHO jurisdictions occupying a shared media market. They explained that having consistent messaging and policies in the region was important, and that ABAHO was particularly valuable for coming to consensus on different issues. When LHDs’ policies differed, health officers had the information to explain why those differences existed. 

“One of the main benefits of ABAHO is it really leverages the competencies and skill sets of each of the individual counties and their resources to enhance all of the counties’ ability to respond...[and] having a forum in which health officers – health departments – can discuss this, look at the surveillance data, look at our local reality and come to, if not a consensus, an agreed upon way of approaching something and a rationale if there are differences”.

### The Association of Bay Area Health Officials Pandemic Influenza Planning Work Group

The health officers and deputy health officers participating in the study were asked about the strengths and weaknesses of the ABAHO Pandemic Influenza Planning Work Group that was formed in 2006. One notable benefit of the planning process was that the group produced many products. One product was a matrix of response decisions stratified by pandemic stage that would guide the discussion of preparedness and response across jurisdictions. One respondent explained the value of this particular tool, 

“We had done so much work, so much to the point that we actually had a grid... that actually has decisions for various stages for various measures that could be implemented. Everything from surveillance to isolation and quarantine to school closure, along with some standard or templates for orders, for school closure... If we hadn’t created [that] ahead of time we’d have to invent it on the fly... It helped us to really understand and have debates on why we’d be doing certain things and help all opinions sort of get out there and come to some bit of consensus.”

It was this type of rigorous, scenario-driven planning that helped health officials and other key decision-makers become familiar with the challenges of addressing an influenza pandemic prior to the arrival of novel H1N1. The Work Group, now called the ABAHO Public Health Preparedness Subcommittee, has since broadened its focus to all-hazards events. Several interviewees expressed a desire to see this type of work continue, noting how valuable it proved to be in laying the groundwork for the response to the H1N1 pandemic.

### Regional communication about epidemiology issues

Currently, most epidemiologists and many health officers rely heavily on informal networks for communicating about epidemiology and surveillance issues and cited using these networks during the H1N1 pandemic. These networks included professional groups, such as the California Conference of Local Health Data Managers (CCLHDM); workgroups sponsored by organizations such as the National Association of County and City Health Officials (NACCHO), the Council of State and Territorial Epidemiologists (CSTE), and the Association of Public Health Laboratories (APHL); epidemiology-specific web-based Yahoo and Google groups; and peers in similar job positions.

Many respondents, particularly epidemiologists, indicated that they would like to have more regional communication about epidemiology issues. Topics of interest included sharing tools, resources, forms, statistical code and analysis, best practices, use of ICS, developing memoranda of understanding, and networking. 

“I think it would be a great benefit to have some venue, even if it was twice a year, to put epidemiologists regionally in a room together and present a few things and hash out a few things and talk informally... there are so many things that, as epidemiologists, we do in every county that literally one person – some central person – could actually do way more efficiently.”

“We had a meeting recently as part of our regional group where the [epidemiologists] got together and our task was to discuss how to make epidemiology regional...we decided there were a lot of ways that we can help each other in terms of...data analysis, like if you’ve already written [statistical] code for example. If you already have a database set up. If we have counties...in this region, counties that don’t have epidemiologists, exploring how we can help.”

One respondent noted that efforts to share tools and information among epidemiologists need to increase due to the fact that epidemiology capacity in local health departments in the region is declining. 

“Of concern is that we seem to be losing epidemiologists from our local health department....I think for the counties that do have epidemiologists that our ability to provide mutual aid to other counties is limited because of our job duties and also because of our current funding streams.”

## Discussion

To our knowledge, this is the first study conducted with ABAHO, representatives from a group of LHDs in the San Francisco Bay Area, that describes how each LHD responded and worked with other LHDs in the region during an infectious disease emergency. Other studies that have reviewed various aspects of the 2009 novel influenza A (H1N1) experience (e.g., public health surveillance activities, field investigations, and laboratory considerations) have been published [24-30]; the current study adds to this body of knowledge by describing key aspects of LHDs’ epidemiology and surveillance responses, including resource management, regional communication, and how LHDs worked with one another during the response. In addition, this study updates findings from a previous study that examined local variation in public health preparedness among a sample of LHDs in California [31]. Epidemiologic investigations, laboratory activities, and public health surveillance are core public health activities for protecting communities from infectious diseases [6]. With the emergence of a novel virus in human populations, many questions arose regarding the epidemiologic features of infection with novel influenza A (H1N1), such as the severity of illness (case-fatality proportion, rate of hospitalization, etc.), the transmissibility of the virus, characteristics associated with infection and adverse health outcomes [32]. The epidemiology and surveillance response to novel influenza A (H1N1) was important for gathering the necessary information to answer the key epidemiologic questions regarding the novel influenza virus [33].

In order to understand LHD responses during the pandemic, it was important to understand the context in which these public health systems operated. We conducted semi-structured interviews with key informants of the LHDs, collecting in-depth information about the perceptions and experiences of public health professionals responding to a novel virus, including details about the complex processes outlined in our conceptual framework (Figure 1). The interviews enabled the research staff to engage local partners in order to collect potentially sensitive information regarding lessons learned, organizational performance, public health staffing and positions, leadership, political considerations, organizational history, and the “culture” of their organizations. This engagement with local partners was crucial for building trust between the research staff and the LHD professionals in order to obtain the information needed for the study. The research staff also reviewed documents about organizational activities in order to augment the information obtained in the interviews. This study revealed how each organization utilized information from the environment (such as collecting various sources of data and communicating with public health professionals from other LHDs) in order to make the best public health decisions during an infectious disease emergency.

The study highlights the benefits of prior planning and preparedness activities on organizational response and communication during an infectious disease emergency. Since 2006, there has been considerable investment devoted to strengthening and maintaining public health systems for a response to pandemic influenza; ABAHO started its own pandemic preparedness planning as well [18]. However, the emergence of novel influenza A (H1N1) virus among humans in April 2009 caught the world by surprise. For years, public health professionals had been preparing for a scenario in which influenza A (H5N1) would emerge from Southeast Asia [34,35], only to confront instead a novel influenza A (H1N1) virus of swine origin in San Diego County, California [7,8]. Pandemic planning scenarios assumed a “worst case scenario” with a high level of illness severity, with some time from recognition of the pandemic to widespread geographic dissemination [35]. Although the planning scenarios differed from the actual events in 2009, the public health efforts that were initiated prior to the 2009 pandemic had substantial benefits that were not fully realized until 2009. Previous regional planning activities for public health emergencies (including pandemic influenza) among a group of LHDs in the San Francisco Bay Area were crucial for communication and coordination of the public health response to novel influenza A (H1N1) in 2009.

In addition, the study revealed many aspects of the epidemiology and surveillance response among LHDs that have implications for public health preparedness and emergency response training, public health best practices, regional public health collaboration, and information sharing. The public health workforce’s experiences with these activities are critical for strengthening public health systems and incorporating lessons learned into the planning process in order to improve the response to future infectious disease outbreaks and other public health emergencies. The study showed that among the 12 LHDs, case reports, reporting of hospitalized and fatal cases, and laboratory data were crucial sources for situation awareness, with the use of emergency room or outpatient data supplementing the majority of the agencies’ information. Perhaps even more importantly, the interview data revealed the many challenges to collecting school absenteeism data during the initial phases of the pandemic, including a lack of information-sharing agreements with local offices of education; lack of standardization and automation in these data systems; and incomplete collection of the data. If these data sources prove to be useful for monitoring a given public health situation, steps should be taken to address these problems and strengthen the relationship between public health agencies and school systems.

Several areas of successful collaboration among LHDs were identified, including the sharing of laboratory capacity, regional preparedness and response planning, and public messaging from public health leaders. The study found that there is a widely expressed desire for more information sharing among public health professionals involved in epidemiology and surveillance functions in the region. Given the success of regional collaboration in the aforementioned areas, improved regional communication about epidemiology and surveillance issues may help to strengthen regional preparedness, decrease inefficiencies [31,36], and provide support for epidemiology functions at a time when many public health agencies are facing budget cuts [37] or other challenges to maintaining epidemiology capacity.

While the many experiences and perceptions described in this study may not be unique to LHDs in the San Francisco Bay Area, the research study describes the individual local response to novel influenza A (H1N1) as well as how a region of local health jurisdictions – a region that had planned together – worked together during an actual response. When faced with an emerging pandemic in an environment with limited resources, many public health professionals commented that having the ability to communicate about key aspects of the response across jurisdictional boundaries was critical to their response. Perhaps the greatest concern, as expressed by a key informant, pertains to the possible decline in organizational capacity due to funding cuts in many local health departments [38]. Cuts in public health preparedness funding undermine the ability of public health departments to maintain the infrastructure needed to carry out core public health activities, including epidemiology and public health surveillance activities. With many LHDs experiencing additional budget cuts since this study was conducted, the region may have less capacity now than it had previously which would adversely affect the region’s ability to respond to a future event.

The EpiNet study is subject to several limitations. First, the results of this study are not necessarily generalizable to other LHDs, due to the varying size, staffing levels, and demographic characteristics of the populations in the San Francisco Bay Area. In addition, it is unlikely that these results will be generalizable to responses to other infectious disease emergencies, given the unique circumstances of the H1N1 pandemic. Nevertheless, the study uncovered key aspects of the epidemiology and surveillance response of LHDs in the San Francisco Bay Area that will help strengthen the response to a future event. Second, there may be additional individuals within each organization (e.g., laboratory personnel) or individuals from collaborating organizations (e.g., hospitals, schools, or CDPH) who could have provided additional insights into the public health responses of LHDs. CDPH played a crucial role in providing overall guidance to and coordination of activities during the response, in addition to characterizing important aspects of the epidemiology of the novel virus, such as factors (e.g., obesity [39]) associated with death or hospitalization [9,40] and severity of illness among pregnant and postpartum women [41,42] and children [43]. However, CDPH had a different role from LHDs in California. With limited research resources, the EpiNet Study focused on the epidemiology and surveillance response of LHDs in the San Francisco Bay Area and did not include key informants from CDPH. While the EpiNet study examined many aspects of the response to the H1N1 pandemic, including sources of epidemiological data and the use of ICS, the study team did not seek to evaluate the LHD responses to the pandemic. The study was not equipped to evaluate the strengths of various data sources gathered or variations of the ICS structure employed. In fact, the study team was able to obtain access to and trust of many key informants because we made explicit that LHDs would not be evaluated. Finally, the data were collected by self report through key informant interviews; the data collection methods did not include personal observations of organizational performance. Despite these limitations, the information obtained and the lessons learned in this study should be helpful in discussions of how to strengthen overall preparedness in the region, recognizing that the pandemic context had many features that were unique to its circumstances.

## Conclusions

The study demonstrated that among the 12 LHDs, case reports, reports of hospitalized and fatal cases, and laboratory data were crucial sources for situation awareness, with the use of emergency room or outpatient data supplementing the majority of the agencies’ information. Perhaps even more importantly, the pandemic revealed the many challenges to collecting school absenteeism data, including a lack of information-sharing agreements with local offices of education, lack of standardization and automation in these data systems, and incomplete collection of the data. Steps should be taken to address these issues and strengthen the relationship between public health agencies and school systems. The study reviewed many aspects of the epidemiology and surveillance response among LHDs that have implications for public health preparedness and emergency response training, public health best practices, regional public health collaboration, and the perceived need for information sharing. The public health workforce’s experiences with these activities should inform the process of strengthening public health systems and incorporating lessons learned into planning for future infectious disease outbreaks and other public health emergencies.

## Abbreviations

AAR: After-Action Report; ABAHO: Association of Bay Area Health Officials; APHL: Association of Public Health Laboratories; CCLHDM: California Conference of Local Health Data Managers; CDC: Centers for Disease Control and Prevention; CDPH: California Department of Public Health; CSTE: Council of State and Territorial Epidemiologists; DOC: Departmental Operations Center; EpiNet: Epidemiology Networks in Action; ICS: Incident Command System; ILI: Influenza-like Illness; JITT: Just-In-Time Training; LHD: Local Health Department; MRC: Medical Reserve Corps; NACCHO: National Association of County and City Health Officials; RT-PCR: Reverse-Transcriptase Polymerase Chain Reaction.

## Competing interests

The authors declare that they have no competing interests.

## Authors’ contributions

WTAE and TJA designed the study. WTAE, AWC, JB conducted the key informant interviews. The transcripts were analyzed by AWC, JF, WTAE, and WT. The initial manuscript was drafted by WTAE and AWC. All authors read, contributed to, and approved the final manuscript.

## Pre-publication history

The pre-publication history for this paper can be accessed here:

http://www.biomedcentral.com/1471-2458/13/276/prepub
